# Computational Insights and In Vitro Validation of Antibacterial Potential of Shikimate Pathway-Derived Phenolic Acids as NorA Efflux Pump Inhibitors

**DOI:** 10.3390/molecules27082601

**Published:** 2022-04-18

**Authors:** Karishma Singh, Roger M. Coopoosamy, Njabulo J. Gumede, Saheed Sabiu

**Affiliations:** 1 Department of Biotechnology and Food Science, Faculty of Applied Sciences, Durban University of Technology, P.O. Box 1334, Durban 4000, South Africa; karishmas@dut.ac.za; 2 Department of Nature Conservation, Faculty of Natural Sciences, Mangosuthu University of Technology, P.O. Box 12363, Durban 4026, South Africa; rogerc@mut.ac.za; 3 Department of Chemistry, Faculty of Natural Sciences, Mangosuthu University of Technology, P.O. Box 12363, Durban 4026, South Africa; ngumede@mut.ac.za

**Keywords:** ciprofloxacin, combination therapy, efflux pumps, phenolic acids, NorA

## Abstract

The expression of the efflux pump systems is the most important mechanism of antibiotic resistance in bacteria, as it contributes to reduced concentration and the subsequent inactivity of administered antibiotics. NorA is one of the most studied antibacterial targets used as a model for efflux-mediated resistance. The present study evaluated shikimate pathway-derived phenolic acids against NorA (PDB ID: 1PW4) as a druggable target in antibacterial therapy using in silico modelling and in vitro methods. Of the 22 compounds evaluated, sinapic acid (−9.0 kcal/mol) and p-coumaric acid (−6.3 kcal/mol) had the best and most prominent affinity for NorA relative to ciprofloxacin, a reference standard (−4.9 kcal/mol). A further probe into the structural stability and flexibility of the resulting NorA-phenolic acids complexes through molecular dynamic simulations over a 100 ns period revealed p-coumaric acid as the best inhibitor of NorA relative to the reference standard. In addition, both phenolic acids formed H-bonds with TYR 76, a crucial residue implicated in NorA efflux pump inhibition. Furthermore, the phenolic acids demonstrated favourable drug likeliness and conformed to Lipinski’s rule of five for ADME properties. For the in vitro evaluation, the phenolic acids had MIC values in the range 31.2 to 62.5 μg/mL against *S. aureus,* and *E. coli*, and there was an overall reduction in MIC following their combination with ciprofloxacin. Taken together, the findings from both the in silico and in vitro evaluations in this study have demonstrated high affinity of p-coumaric acid towards NorA and could be suggestive of its exploration as a novel NorA efflux pump inhibitor.

## 1. Introduction

Globally, high rates of morbidity and mortality have been partly linked to microbial infections, with some highly resistant pathogenic bacteria (*Enterococcus faecium, Staphylococcus aureus, Klebsiella pneumoniae, Acinetobacter baumannii, Pseudomonas aeruginosa,* and *Enterobacter* spp.) frequently implicated [1]. These microorganisms are receiving priority research attention with a view to improve existing antibiotics as well as develop novel and efficient drug candidates [1,2]. Expression of the efflux pump systems (EPS) is the most important mechanism of antibiotic resistance in bacteria, as it contributes to reduced concentration and the subsequent inactivity of administered antibiotics [2]. The efflux pumps (EP) are membrane-spanning proteins found in both prokaryotic and eukaryotic cells [2,3] and studies have linked antimicrobial resistance (AMR) to alterations on the structural architecture of EPs in Gram-positive and Gram-negative bacteria [3,4]. The NorA system, which is a 388 amino acid protein with 12 transmembrane segments (TMS) and a member of the bacterial secondary transporter major facilitator superfamily (MFS), remains the most studied EP in efflux-mediated resistance in *S. aureus* and *E. coli* [4,5,6]. Over the years, studies have lent credence to NorA as an attractive druggable target for antibacterials and plant secondary metabolites such as phenolic acids which have been identified as potential NorA efflux pump inhibitors (EPIs) [3,4,5,6,7,8]. Such EPIs facilitate increased intracellular concentrations of antibiotics which in turn restored cell susceptibility and antibacterial activity of the antibiotic [3,4,5,6,7,8]. Despite the evaluation and subsequent identification of phenolic acids as NorA inhibitors, the exact mechanism of their inhibitory action is still largely unknown. More importantly, their possible combination with conventional antibiotics to strengthen their antibacterial potency remains underexplored and necessitates the need for more action-guided studies focusing on their mechanism of action and possibility of combination therapy. Hence, the present study investigated the molecular mechanism of interactions between the NorA efflux pump and shikimate pathway-derived phenolic acids using in silico and in vitro experimental models. The data obtained from this study is vital to repurposing phenolic acids as novel NorA inhibitors through the establishment of their molecular interactions with the active site amino acid residues of NorA.

## 2. Results and Discussion

### 2.1. In Silico Studies

#### 2.1.1. Molecular Docking

Molecular docking measures the affinity and pose of compounds at the active site of an enzyme or receptor, with a higher negative binding score indicating a better posed compound [9]. In this study, of the 22 shikimate pathway-derived phenolic acids docked against the NorA efflux pump, sinapic acid and p-coumaric acid had the best affinity towards the protein (Supplementary Table S1). The binding of sinapic acid to NorA yielded five poses, with the top scoring pose having an affinity score of −9.04 kcal/mol, an e-model score of −42.02 kcal/mol and an IFD score of −771.92 kcal/mol (Table 1). The p-coumaric acid-NorA complex also had five poses with the top scoring pose having docking, e-model and IFD scores of −6.91, −39.45 and −767.95 kcal/mol, respectively (Table 1). These scores were higher than that of the top scoring pose for ciprofloxacin which exhibited a docking score of −4.31 kcal/mol, an e-model score of −59.19 kcal/mol and an IFD score of −760.89 kcal/mol (Table 1). Generally, the higher negative binding scores observed with sinapic acid and p-coumaric acid relative to ciprofloxacin could be indicative of their better affinity towards NorA. The observation regarding binding scores in this study is consistent with previous reports [5,6,7,8,10,11,12], where natural compounds with higher docking scores showed higher binding affinity towards NorA.

**Figure 1:**
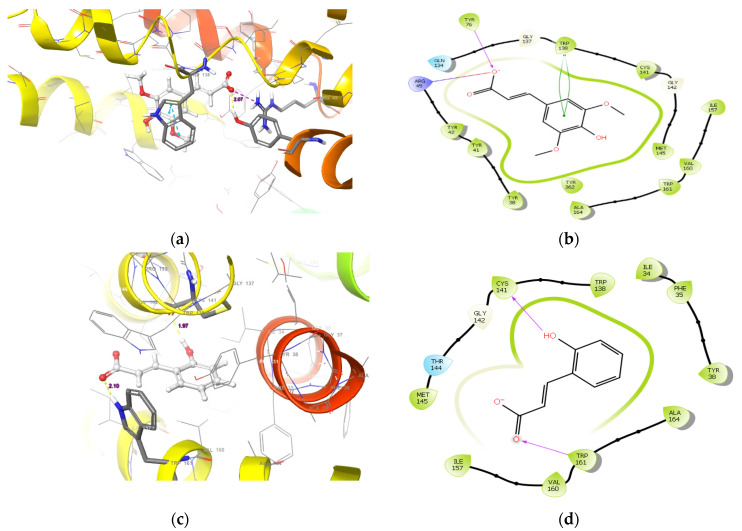

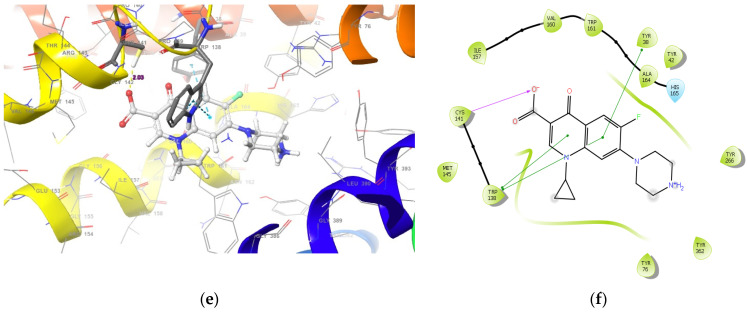
The binding mode of (**a**) sinapic acid (**c**) p-coumaric acid, and (**e**) ciprofloxacin in the active site cavity of NorA. The ligand interaction diagram of (**b**) sinapic acid, (**d**) p-coumaric acid, and (**f**) ciprofloxacin.

A visual inspection of the top scoring pose of sinapic acid revealed a hydrogen bond between the OH group of Tyr76 and the carboxylate moiety of sinapic acid with a bond radius of 2.07 Å (Figure 1a,b). Furthermore, a salt bridge interaction between Arg45 and the carboxylate moiety was also observed. Additionally, a π-π interaction between the benzimidazole moiety of Trp138 and 2,6-dimethoxyphenol moiety was observed (Figure 1b). Unlike with sinapic acid, a hydrogen bond with a radius of 1.97 Å was observed between the carbonyl group of Cys141 and the OH group of p-coumaric acid alongside another hydrogen bond between the NH group of Trp161′s benzimidazole moiety and its carbonyl group (Figure 1c,d). Furthermore, visual inspection reveals a hydrogen bond between Cys141 and a carboxylate moiety of ciprofloxacin; and a bond radius of 2.03 Å (Figure 1e,f). An interesting π-π interaction network is observed between the benzimidazole moiety of Trp138 and the quinoline moiety of ciprofloxacin that extends to the phenol ring of Tyr38 (Figure 1e,f). Such interactions elicited by both sinapic acid and p-coumaric acid at the binding pocket of NorA could be indicative of their higher and better affinities for the protein [13,14,15]. More importantly, the different binding modes exhibited by the two compounds and the standard tested by IFD could be attributed to their diverse structures, as it was evident that the binding mode of ciprofloxacin was very distinct from the two phenolic acids since it belongs to a different chemical class of compounds.

#### 2.1.2. Molecular Dynamics

**Figure 2:**
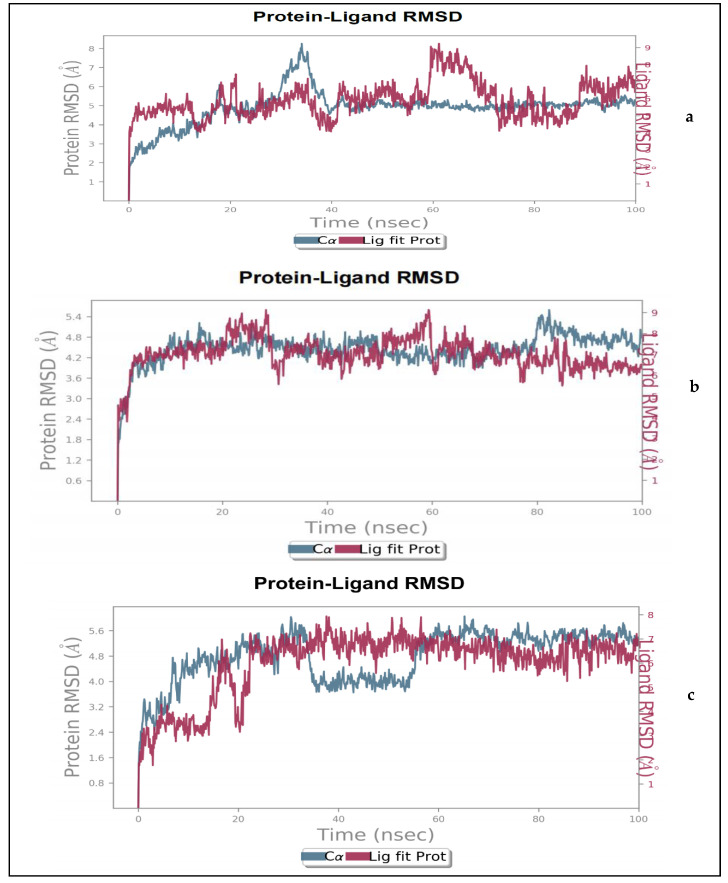
Protein-ligand root mean square deviation for NorA with (**a**) sinapic acid, (**b**) p-coumaric acid and (**c**) ciprofloxacin in a simulation trajectory of 100 ns.

**Figure 3:**
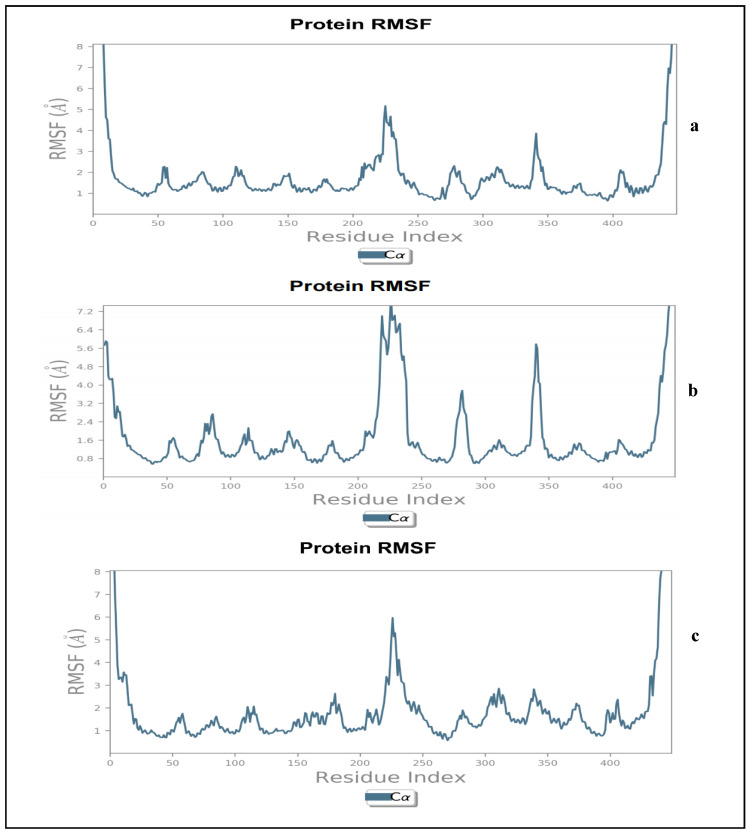
Protein root mean square fluctuation plot for NorA in the presence of (**a**) Sinapic acid, (**b**) p-Coumaric acid, and (**c**) Ciprofloxacin as complexes.

Since docking is only a preliminary indication of the investigated compounds’ fitness within the active site of NorA, the binding poses of both phenolic acids were further subjected to molecular dynamics simulations (MDS), which allowed for analysis of the physical movements and interactions of atoms and molecules of a system. The data obtained with respect to the post-dynamic analyses of the compounds presented as root mean square deviation (RMSD) and root mean square fluctuations (RMSF) are shown in Figure 2 and Figure 3, respectively. The degree of stability and convergence of a ligand-protein system is normally measured by RMSD of the protein-ligand complex, and protein RMSF and their lower values determine how well the system is equilibrated and stabilized during the simulation window [15,16,17]. In this study, it could be observed that NorA of the sinapic acid-NorA complex underwent some conformational changes from 0–40 ns with an average RMSD value of 4.50 Å but changed to 7 Å around 29–37 ns and subsequently equilibrated with an average RMSD of 5.10 Å from 40 ns and throughout the entire simulation period (Figure 2a). Sinapic acid on the other hand, had an average RMSD of 5.8 Å during the simulation time from 0–58 ns, with no pronounced conformational change. However, obvious changes were noticed around 59–71 ns (Figure 2a). Figure 2b shows the evolution of the RMSD of NorA with respect to the reference frame in a simulation window of 0–100 ns. The protein system is greatly equilibrated with no major fluctuations of the protein during the simulation window, having an average RMSD of 4.65 Å. Typically, p-coumaric acid-NorA complex was the most stable system with the lowest RMSD value and only slightly diffused from the apo-protein after 80 ns, but the system was equilibrated prior to this (Figure 2b). This was, however, in sharp contrast with the observations with sinapic acid (Figure 2a) and ciprofloxacin (Figure 2c), where the extent of deviation from the apo-NorA were relatively higher with the highest observed with sinapic acid. Ciprofloxacin- NorA complex had an average RMSD of 4.5 Å over 0–38 ns MDS period followed by a drop in RMSD between 35–55 ns, attributable to the adoption of a different and more favorable conformational change with an average RMSD of 3.90 Å and finally stabilized beyond 55 ns throughout the simulation period with an average RMSD of 5.20 Å. These observations regarding RMSD in this study is consistent with previous studies [17,18], where greater stability of the ligand-protein complexes was attributable to lower RMSD values. Furthermore, these observations above highlight the fact that it is important to choose the correct binding pose when deciding on the selection of compounds for further studies. There is a large body of evidence suggesting that pose selection is important in hit identification in drug discovery [12,13], and there is no direct way to overcome the challenge of selecting the correct binding poses. However, scientists have successfully used MD simulation [12,13,14,15,16] in post-processing method to overcome this challenge. Furthermore, scientists at Schrödinger in 2020 have introduced a robust IFD-MD premium module to overcome this challenge, which broadens this method’s domain of applicability. In fact, prospective and retrospective studies have revealed that this method has achieved the accuracy of predicting the correct binding modes similar to native conformers sought by X-ray crystallography and the Cryo-Electron Microscope [16].

A further probe into the degree of structural flexibility of the protein-ligand complexes measured as RMSF revealed that the sinapic acid-NorA complex (Figure 3a) had more pronounced fluctuations with Asp229, Lys234 and Gly345 with an RMSF of 4.39 Å, 3.70 Å, and 3.86 Å, respectively. The remaining amino acids residues had lesser fluctuations with RMSF ranging between 1.05–2.3 Å (Figure 3a). Contrary to this observation, the p-coumaric acid-NorA complex had pronounced fluctuations with Lys6, Lys11, Pro15, Glu222, Tyr231, Gln237, Phe286, and Ala344 (Figure 3b). These amino acids exhibited RMSF of 5.91, 3.86, 3.08, 5.65, 7.00, 6.81, 6.68, and 3.76 Å, respectively, while other residues had average RMSF values of 0.69–1.09 Å (Figure 3b). Figure 3c depicts the ciprofloxacin-NorA complex. The amino acids that fluctuate more during simulation includes Pro15, Asp226, Asp230, Ala235, and Ile436 with RMSF values of 3.57, 3.24, 5.97, 4.12, and 3.40 Å, respectively. The amino acids that were stable and fluctuated less during simulation had an average RMSF of 0.80–1.9 Å. Generally, high fluctuations corresponding to RMSF values are indicative of more flexibility and less stable bonds, whereas less fluctuations indicates well-structured regions in the complex and less distinct (more stable bonds) [16,17]. Studies have reported that higher peaks are indicative of the amino acid residues that fluctuates the most at the active site, thus resulting in less stable bonds between the ligands and amino acids of the protein [17,18,19]. In the present study, while all the systems displayed more flexible residues (i.e., higher RMSF values) at residue number 200–250; 340–360, it was apparent that p-coumaric acid-NorA complex (Figure 3b) had lesser residue motility at the active sites, which indicates stable bonds between itself and NorA in comparison to sinapic acid-NorA complex (Figure 3a) and ciprofloxacin-NorA complex (Figure 3c). Such lesser residue movement as observed with p-coumaric acid contributes to its structural stability with NorA, which is consistent with the observation with RMSD in this study, where it was found to be most stable in comparison to sinapic acid-NorA and ciprofloxacin-NorA complexes. The RMSF value for p-coumaric acid in this study corroborates previous studies where a similar pattern of fluctuations was observed with the essential amino acid residues at the active site of NorA [9,20,21].

**Figure 4:**
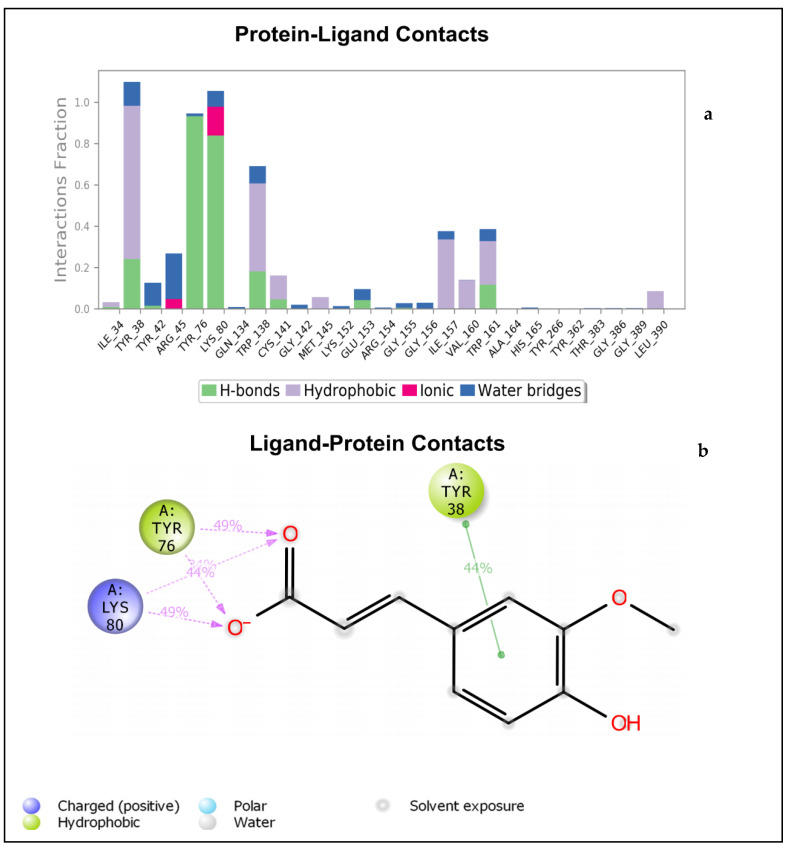
(**a**) Bar chart plot detailing protein-ligand interactions of sinapic acid-NorA complex with the key amino acid residues at NorA active site. (**b**) Simulation interaction diagram detailing the interaction of sinapic acid with NorA that occur in 30% of simulation time from 0–100 ns.

The bar chart in Figure 4a details the contacts made by sinapic acid on the amino acid residues of NorA. It can be noticed that the active site of NorA is essentially hydrophobic. The Tyr76 occurred as the hydrophobic amino acid of NorA involved in hydrogen bonding with the carboxylate moiety of sinapic acid (Figure 4a,b). This hydrogen bond was also captured by IFD (Figure 1a,b). Furthermore, the Tyr38 involved in π-π interaction with sinapic acid and the Lys80 hy-drogen bonds with the carboxylate moiety of sinapic acid was not captured by IFD (Figure 4b); this may be due to the conformational changes in the sinapic acid-NorA complex over the simulation period. Interestingly, a further insight into validation of contacts established with sinapic acid revealed that Tyr76, Lys80, and Tyr38 had more contacts with sinapic acid during the simulation trajectory (Supplementary Figure S1). Figure S1 details the timeline representation of the contacts sinapic acid made with NorA. The dark orange shades indicate the amino acid that had more contacts with sinapic acid, while the lighter orange shades indicate the amino acids that had some contacts with sinapic acid., whereas the amino acids with no orange shades indicates amino acids that had no contacts with sinapic acid. Furthermore, the contact between Tyr38 and sinapic acid diminished (light orange shades) throughout the simulation trajectory, indicating that the ligand had undergone some conformational changes (Supplementary Figure S1).

**Figure 5:**
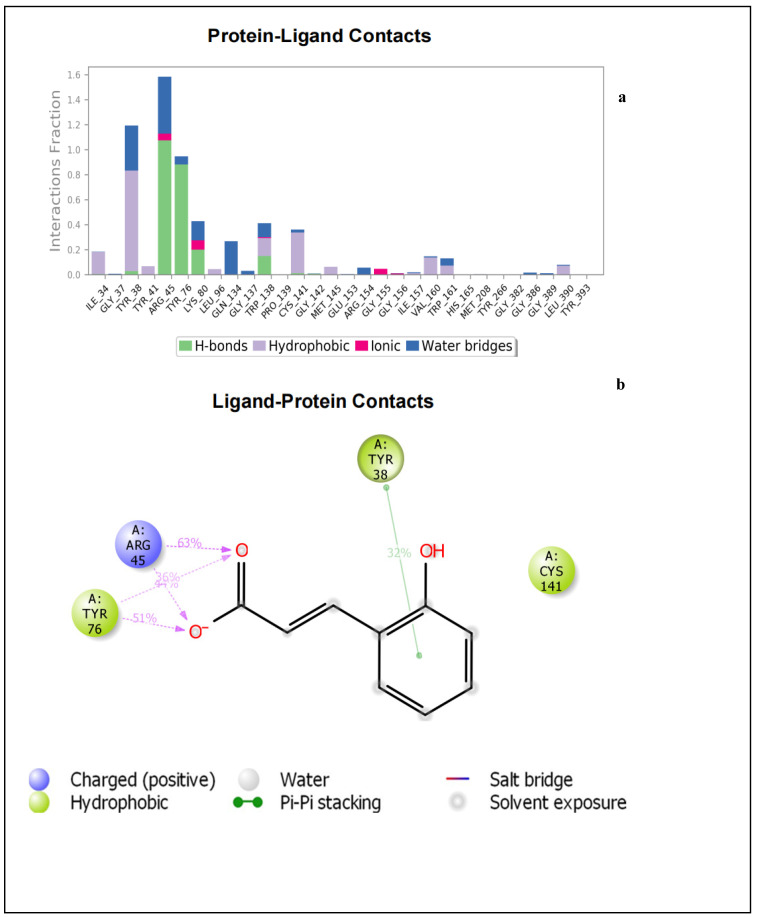
(**a**) Bar chart plot detailing protein-ligand interactions of p-coumaric acid-NorA complex with the key amino acid residues at NorA active site. (**b**) Simulation interaction diagram detailing the interaction of p-coumaric acid with NorA from 0–100 ns.

**Figure 6:**
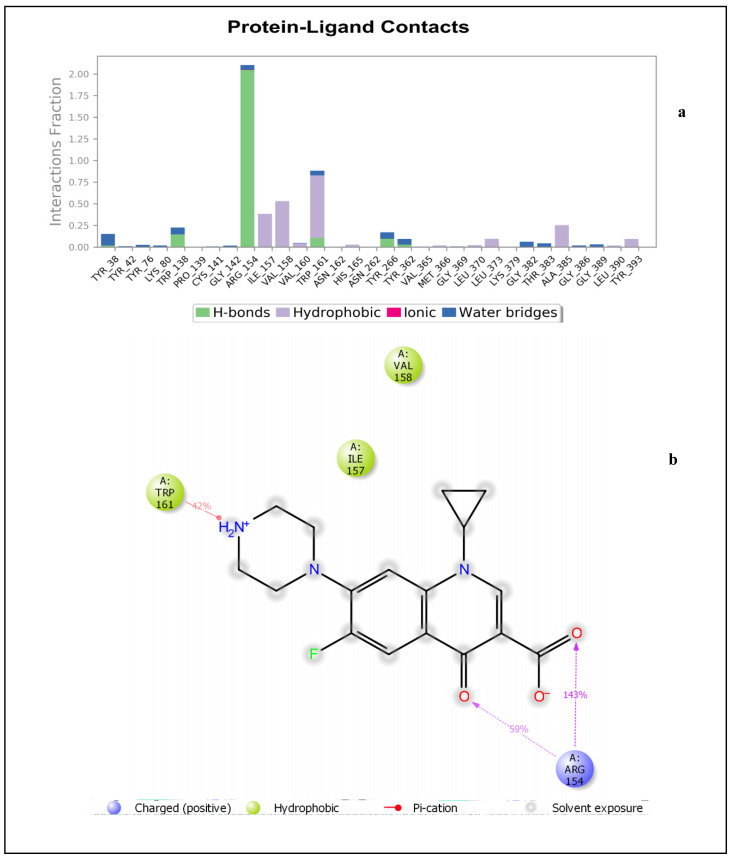
(**a**) Bar chart plot detailing protein-ligand interactions of ciprofloxacin-NorA complex with the key amino acid residues in the active site cavity. (**b**) Simulation interaction diagram detailing the interaction of ciprofloxacin with NorA that occur in 30% of simulation time from 0–100 ns.

Figure 5a depicts the specific NorA amino acids involved in hydrogen bonds, hydrophobic interactions, and water bridges with the p-coumaric acid. This also includes the percentage fraction of these contacts. Figure 5b shows the hydrogen bonds interaction between Tyr76 as well as Arg45 and the carboxylate moiety of p-coumaric acid. Tyr38 shows a π-π interaction with the phenol moiety of p-coumaric acid. This binding mode was not captured by IFD (Figure 1d), meaning that the backbone of the enzyme had undergone some conformational changes. The details of the timeline representation of the contacts that existed between p-coumaric acid and NorA is presented in (Supplementary Figure S2). Arg45 has more pronounced contacts with the ligand (dark orange shades), whereas Tyr48 has some dark orange and orange shades, indicating that it is the second most common amino acid that made contacts with p-coumaric acid (Supplementary Figure S2). It could also be seen that Tyr76 exhibits orange shades, indicating that the amino acid made some contacts with p-coumaric acid and it is noteworthy that the hydrogen bond exhibited by Cys141 and Trp138 (observed in the IFD pose) was observed at the beginning of the simulation time plot and diminished throughout the progress of the simulation window. This confirms that the ligand and the receptor adopt a change in conformation during simulation. Just like sinapic acid and p-coumaric acid, ciprofloxacin interacted with NorA through hydrogen bonding and hydrophobic interactions throughout the simulation time of 0–100 ns (Figure 6a). As such, the hydrogen bond between Arg154 and the carboxylate moiety of ciprofloxacin occurs more than 100% of the time (Figure 6a,b). Also, Arg154 hydrogen bonds with the carbonyl group of the quinoline moiety. There is also a π-cationic interaction between Arg154 and the NH^+^ group of the piperazine moiety of ciprofloxacin. The active site cavity has some buried waters (Figure 6b). The fact that these interactions were not captured by IFD could be suggestive of conformational changes illustrative of a different binding mode being obtained by MDS, and this was further validated (Supplementary Figure S3), and it was revealed that the contact between Tyr138 and ciprofloxacin was observed between 0–17 ns. This contact was then lost after 17–100 ns of simulation time, indicating that there was a conformational change between ciprofloxacin and NorA. Furthermore, a more pronounced contact between ciprofloxacin and Arg154 (dark orange shades) was observed from 20–100 ns of simulation time (Supplementary Figure S3), while another contact (light orange shades) was observed between Trp161 and ciprofloxacin that was present from 0–100 ns. Additionally, from the total contacts exhibited by the ciprofloxacin during the simulation window, it was evident that from 0–20 ns the number of contacts fluctuated more, and from 20–100 ns the system attained equilibration, suggesting the occurrence of conformational changes (Supplementary Figure S3). Put together, of the interactions established between sinapic acid and p-coumaric acid towards NorA in this study, the hydrogen bond with Tyr76 of NorA appeared to be crucially common, and its occurrence is consistent with the report of Palazzotti et al. [5], where the occupancy of Tyr76 at the active site of NorA was suggested to be important for the structural stability of the NorA complex.

#### 2.1.3. Pharmacokinetic Properties and Toxicity

The Lipinski’s rule of five has been widely accepted as a physicochemical descriptor for predicting the drug-likeliness of a compound that determines the oral bioactivity of a compound or drug [20,21]. According to Lipinski’s rule of 5, a compound or drug with high drug-likeliness should fulfill the following criteria: molecular mass should not exceed 500 g/mol, hydrogen bond donors (≤5), hydrogen bond acceptors (≤10) and iLogP (<5). In this study, sinapic acid and p-coumaric acid conformed with Lipinski’s rule of five and can be orally administered with the propensity to penetrate the cell wall and reach the NorA active site (Table 2). The fact that the partition coefficient (iLogP) of the phenolic acids was less than 5 is an indication that they are not too hydrophobic and will be able to pass through the bloodstream [19]. Drug absorption in the gastrointestinal tract (GIT) is critical for maintaining optimal plasma concentrations and delivering drugs to the active site with the necessary concentration for maximum therapeutic effects [21]. Sinapic acid, p-coumaric acid and ciprofloxacin showed a high absorption rate in the GIT and data relating to ADME revealed that the two compounds will have higher concentrations at their active site and eventually exert a significant effect when considered as antibacterial agents (Table 2).

Furthermore, the test compounds were non-inhibitors of CYP1A2, CYP2C19, CYP2C9, CYP2D6, CYP3A4 (Table 2), and are less likely to provoke drug-drug interaction when co-administered with other drugs normally metabolized by these CYP isoforms [20,21]. As a result, sinapic acid and p-coumaric acid are comparable to the standard ciprofloxacin and were predicted to have high bioavailability and met the drug-likeliness criteria and could be further exploited as novel NorA inhibitors. Table 3 reveals the human maximum tolerated dose (LD_50_ mg/kg) for the test compounds and ciprofloxacin. According to Verma et al. [22], compounds with LD_50_ values greater than 500 mg/kg but less than 5000 mg/kg are usually considered orally safe. The results of this study revealed that sinapic acid and p-coumaric are class 4 compounds (Table 3) and can, therefore, be safely administered orally. Assessment of toxicity showed that none of the compounds exhibit serious adverse effects, but sinapic acid and p-coumaric acid showed probable hepatotoxic and immunogenic tendencies.

### 2.2. In Vitro Antibacterial Evaluation

Due to the significant results of the in silico evaluation on the probable inhibitory effect of sinapic acid and p-coumaric acid on NorA, an effort was made to establish the potential of these compounds in vitro against representative NorA-bearing Gram-positive and Gram-negative bacteria, and the results are presented in Table 4. Studies have demonstrated the inhibitory effect of bioactive compounds against the specific activity of EPs including NorAs of bacterial strains in vitro [23,24,25]. In this study, both sinapic acid and p-coumaric acid showed significant antibacterial activity against all the test bacteria with MIC and MBC of 31.25 and 62.50 μg/mL, respectively, against S aureus (Table 4). However, p-coumaric acid had a higher activity (MIC 62.50 μg/mL, MBC 125 μg/mL) against *E. coli*. Ciprofloxacin was the most effective against S. aureus (MIC 7.81 μg/mL, MBC 15.63 μg/mL) and *E. coli* (MIC 15.63 μg/mL, MBC 31.25 μg/mL). The antibacterial activity elicited by both sinapic acid and p-coumaric acid in this study are more or less significant or consistent with previous studies on phenolics against bacterial strains [11,26,27,28,29]. Sanhueza et al. [27] demonstrated the antibacterial potential of vanillic acid, p-coumaric acid, gallic acid, and protocatechuic acid with MIC values ranging from 300 to 3000 μg/mL and 500 to 4000 μg/mL against *S. aureus* and *E. coli*, respectively, while ciprofloxacin maintained an MIC of 1500 μg/mL against both strains. Santos et al. [28], on the other hand showed that caffeic acid and gallic acid had MIC values of <1024 μg/mL against *S. aureus*. The antibacterial activity of ciprofloxacin against S. aureus in this study was in agreement with the report of Zimmermann et al. [29] showing MIC values in the range 7.8 to 500 μg/mL. A compound’s ability to form hydrogen bonds with amino acid residues at the active site of the NorA efflux pump might explain its antibacterial properties [22,23,24] and as such the activity displayed by sinapic acid and p-coumaric acid in this study could be attributed to the bonds including that of hydrogen interaction with Tyr76 of the amino acid residue of NorA as demonstrated in the in silico aspect of this study.

**Figure 7:**
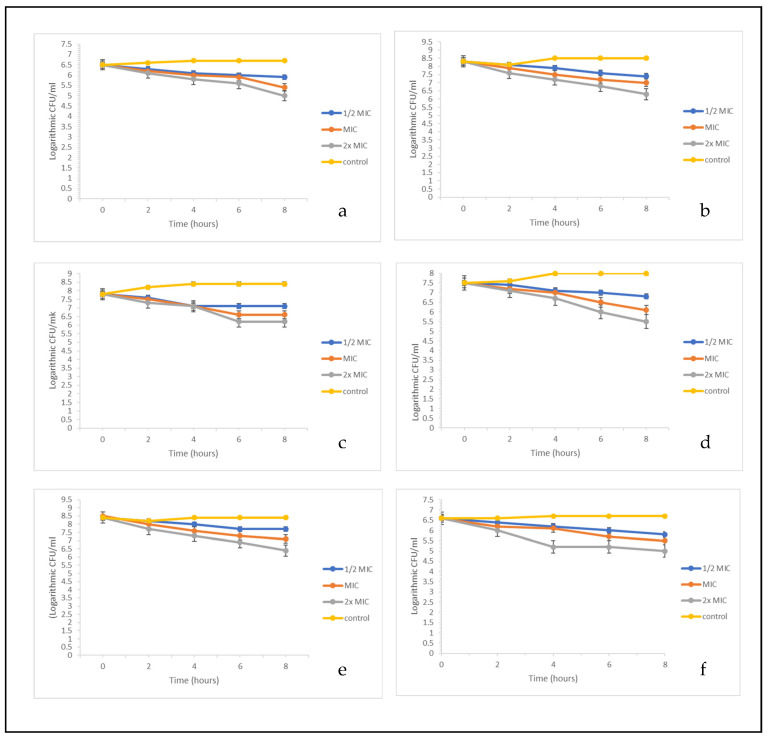
Time-kill kinetics of (**a**) sinapic acid and *Staphylococcus aureus*, (**b**) p-coumaric acid and *S. aureus*, (**c**) ciprofloxacin and *S. aureus*, (**d**) sinapic acid and *Escherichia coli*, (**e**) p-coumaric acid and *E. coli*, (**f**) ciprofloxacin and *E. coli.*

Following demonstration of the antibacterial potential of sinapic acid and p-coumaric acid, further insight was provided into time-kill kinetics of the compounds against the test bacterial strains and the results showed a dose-dependent bactericidal effect (Figure 7). At the investigated concentrations, treatment with sinapic acid (Figure 7a,d), p-coumaric acid (Figure 7b,e), and ciprofloxacin (Figure 7c,f) significantly reduced the number of viable *S. aureus* and *E. coli*. After 8 h of treatment with both compounds and ciprofloxacin, there was a significant decline in bacterial viability relative to the control, with the most prominent period being between 4 to 8 h, and this agrees with previous reports [7,26] on the time-kill kinetics of antimicrobials.

Combination therapy has remained one of the viable strategies to combat multidrug resistant infections, and this has been achieved through the combination of antibiotics with phytocompounds [14,28]. Usually the combined therapeutic agents offer superior antibacterial activity through reduced MIC of the combined compounds and antibiotics [7,23,30]. The results of the combined effect of either sinapic acid or p-coumaric acid with ciprofloxacin in this study was synergistic (FICI ≤ 0.5) against S. aureus, and additive (FICI > 0.5) for sinapic acid against *E. coli* (Table 5). This implies that the phenolic compounds enhanced the antibacterial activity of ciprofloxacin as evidently demonstrated from the reduced MICs (from the respective values in Table 4 to the new values in Table 5) in each case against the test organisms. Previous studies have also demonstrated 4 to 25 times reduction in MIC against the test bacterial cultures following combined treatments with phenolic acids with ciprofloxacin [7,26,31]. On the other hand, the additive effect with sinapic acid against *E. coli* indicates that the combination of ciprofloxacin with sinapic acid exerts an effect greater than the effect of either drug administered individually [14]. Overall, the most remarkable combinations were observed with sinapic acid-ciprofloxacin and p-coumaric-ciprofloxacin against *S. aureus* and *E. coli*, respectively, with FICI of 0.3 each (Table 5).

## 3. Materials and Methods

### 3.1. Molecular Modelling

Schrödinger Life-Sciences Suite version 2021-2 was used for all molecular modelling experiments. Maestro v12.8 [32], a graphical user interface (GUI) was used for visual inspection of the relevant modules such as LigPrep, Protein Preparation Wizard [33], Prime [34,35,36], and Glide [32,37,38,39,40,41] using an Induced Fit Docking (IFD) engine [32,42,43]. The X-ray crystal structure of the NorA efflux pump (PDB: 1PW4 at a resolution of 3.30 Å) was downloaded from the RSCB protein data bank (https://www.rcsb.org/, accessed on 3 June 2021). Protein preparation was performed using default parameter, and interactive optimization was performed with PROPKA at pH 7.4, the resulting structure was minimized with the OPLS4 force-field and convergence was attained at an RMSD of 0.30 Å. All the ligands (22 phenolic acids and ciprofloxacin) were drawn as 2D structures on Maestro v12.8 and were prepared by using LigPrep and ionization including tautomeric states and were estimated by using Epik [32,44,45] at pH 7.4. The OPLS4 force-field was used to generate resulting low energy conformers [32,46,47]. Energy minimization during protein preparation was done using an interactive optimizer and convergence was attained with an OPLS4 force-field, while energy minimization during ligand preparation was performed using OPLS4 force-field [32,42,43,44]. Subsequently, docking at the active site of NorA was executed using the IFD protocol [32,39,45], where the active site is marked by the co-crystalized ligand and the top scoring compounds were selected, used as starting structures and then subjected to a system builder before molecular dynamics (MD) simulation [32,46,47,48]. The MD model system was built on an orthorhombic box utilizing SPC, an explicit solvation model. The model system was neutralized by adding counter ions such as Na^+^ and Cl^−^ ions at a concentration of 0.15 M. The MD simulation time of 100 ns, and a simulation trajectory of 100 ns including 1000 frames for all simulations, were performed. Furthermore, MD simulation was performed in the NPT ensemble at a temperature of 300 K at a pressure of 1.013 bar to monitor the behaviour of the ligand-protein complex, protein stability [root mean square deviation (RMSD)], and conformational fluctuations [root mean square fluctuation (RMSF)] of the ligands at the active site of NorA.

### 3.2. Pharmacokinetic Properties and Toxicity Risk Assessment

The absorption, distribution, metabolism, and excretion (ADME) properties of the top scoring phenolic acids obtained through docking were predicted using the SWISSADME server (http://www.swissadme.ch/, accessed on 15 June 2021), and data relating to molecular properties (H-bond acceptors, H-bond donors, logP, and surface area), absorption (solubility, gastrointestinal absorption (GIT), skin permeability and p-glycoprotein binding), distribution (blood barrier permeability (BBB), metabolism (CYP substrate and inhibitors) and excretion (total clearance and renal transport), were established. Furthermore, the PROTOX II database (https://tox-new.charite.de/, accessed on 15 June 2021) was used to predict probable toxicity profiles of the compounds, and information relating to the ADME toxicity, human maximum tolerated dose, hepatotoxicity, immunotoxicity, carcinogenicity, mutagenicity and cytotoxicity of the compounds were obtained.

### 3.3. In Vitro Antimicrobial Evaluations

#### 3.3.1. Source and Preparation of Bacterial Cultures

The bacterial cultures [*Staphylococcus aureus* (ATCC 29213), and *Escherichia coli* (ATCC 25922)] used in this study were obtained from Anatech Analytical Technology, Olivedale, Gauteng, South Africa. Mueller-Hinton agar was used in all the cultures and the turbidity of all the bacteria was adjusted to 0.5 McFarland standard by selecting three to five well-isolated colonies from agar plate cultures. Further dilution (1000-fold) was performed to obtain an inoculum size of 1 × 10^6^ CFU. The cultures were incubated at 37 °C for 24 h before being compared to a blank in terms of turbidity caused by microbial growth [7].

#### 3.3.2. Test Compounds

The phenolic compounds (sinapic acid and p-coumaric acid, selected based on the in silico results relating to prominent affinity for NorA) and the reference standard (ciprofloxacin) [3,4,5,6,7] procured from Sigma-Aldrich (St. Louis, MO, USA) were dissolved in dimethyl sulphoxide (DMSO) (0.5 mL) and diluted with water (4.5 mL) to make a stock solution of 1000 μg/mL of each compound. To obtain the required concentrations of 500, 250, 125, 62.5, 31.25, 15.63, 7.81, 3.91, 1.95, and 1 μg/mL, further progressive double dilution with Mueller-Hinton (MH) broth was performed. A control test was performed with a test medium supplemented with DMSO at the same dilution as that used in this experiment to ensure that the solvent had no effect on microbial growth.

#### 3.3.3. Determination of Minimum Inhibition Concentration (MIC) and Minimum Bactericidal Concentration (MBC)

The broth micro dilution technique [49], was used to determine the MIC of the test compounds. In a 96-well microtiter plate, 25 μL of MH broth was dispensed in each well followed by the addition of 25 μL each of the prepared sinapic acid, p-coumaric acid and ciprofloxacin, respectively. Exactly 25 μL of each standardized bacterial inoculum (*S. aureus*, and *E. coli*) was then added to all the wells and incubated at 37 °C for 24 h. The MIC was determined as the lowest concentration that resulted in no visible bacterial growth, while the MBC was determined after the MIC was determined by taking 25 μL from each well and sub culturing on freshly prepared nutrient agar plates. The MBC was determined as the lowest concentration where no visible growth was observed after 24 h of incubation at 37 °C [30]. Both the MIC and MBC were carried out in triplicate and control cultures (MH broth only) were prepared for all bacteria.

#### 3.3.4. Time-Kill Assay

To determine the rate of killing of sinapic acid, p-coumaric acid and ciprofloxacin against *S. aureus*, and *E. coli,* a previously reported protocol was used [49]. Treatment concentrations (1/2MIC, MIC, and 2MIC) of the test compounds were prepared and incorporated into 9 mL MH broth. Two controls were prepared: one MH broth without phenolic acids or ciprofloxacin and one with ciprofloxacin. All test tubes received 20 μL of inoculum (1 × 10^5^ CFU/mL) and were incubated at 37 °C. Following a 24-h incubation period at 37 °C, one millilitre aliquots were taken from the test tubes at intervals of 0, 1, 2, 3, 4, 5, 6, and 8 h and aseptically transferred into fresh nutrient agar plates for CFU determination by plate count technique. Following that, the emerging microbial colonies were counted, and CFU/mL was calculated, compared to the controls, and a log CFU/mL graph was plotted against time.

#### 3.3.5. Combination Therapy (Checkerboard Assay)

The checkerboard method [14], with minor modifications, was used to assess the potential interactions between either sinapic acid or p-coumaric acid and ciprofloxacin. In a 96-well microtiter plate, two-fold serial dilution of the stock solution of sinapic acid, p-coumaric acid and ciprofloxacin were done in a 25 µL Mueller-Hinton broth, in decreasing concentrations, respectively. A standardized bacterial suspension of 25 µL of (1 × 10^8^ CFU/mL) was used. The wells containing only single treatments of sinapic acid, p-coumaric acid, and ciprofloxacin served as positive controls. The microtiter plate was incubated at 37 °C for 24 h. Subsequently, the fractional inhibitory concentration index (FICI) was calculated to determine the type of interaction or effect produced by the combination of sinapic acid and ciprofloxacin and p-coumaric acid and ciprofloxacin using the using the expression:

FICI = (MIC of phenolic acid in combined treatment/MIC of phenolic acid alone) + (MIC of ciprofloxacin in combined treatment/MIC of ciprofloxacin alone) [28]. And the FICI for each combination was interpreted as follows: FICI ≤ 0.5 synergistic effect, 0.5 < FICI ≤ 4 additive effect and FICI > 4 antagonistic effect [28].

### 3.4. Statistical Analyses

All the in vitro experiments were performed in triplicate and results are presented as means ± standard deviation. To compare data between groups, one-way ANOVA was used, and *p* ≤ 0.05 was considered significant using online version IBM SPSS Statistics 28 (https://analytivs.olsps.com/spss-statistics/, accessed on 9 October, 2021).

## 4. Conclusions

Of the 22 shikimate pathway-derived phenolic acids computationally evaluated against NorA in this study, only sinapic acid and p-coumaric acid had the best affinity towards the protein and established significant interactions including H-bonds with TYR 76 that is thought to aid in the inhibition of the NorA efflux pump. The two compounds also enhanced the structural stability of their respective complexes with NorA, and this observation was consistent with the in vitro evaluation where sinapic acid and p-coumaric acid elicited significant antibacterial effect against *S. aureus* and *E. coli*. Taken together, the findings from both the in silico and in vitro evaluations in this study demonstrated the high affinity of sinapic acid and p-coumaric acid towards NorA and could be suggestive of their exploration as novel NorA inhibitors that will find practical application in the fight against AMR.

## Figures and Tables

**Table 1:** Molecular docking energetics for sinapic acid, p-coumaric acid and ciprofloxacin after binding with NorA.

No.	Title	Docking Score	Glide Emodel	XP G Score	IFD Score
		(kcal/mol)	(kcal/mol)	(kcal/mol)	(kcal/mol)
1	Sinapic Acid	−9.04	−42.02	−9.04	−771.92
2	Sinapic Acid	−7.27	−38.66	−7.27	−770.04
3	Sinapic Acid	−6.45	−31.35	−6.45	−768.67
4	Sinapic Acid	−2.04	−36.05	−2.04	−765.21
5	Sinapic Acid	−1.21	−35.90	−1.21	−763.33
6	p-Coumaric Acid	−6.91	−39.45	−6.91	−767.95
7	p-Coumaric Acid	−6.32	−40.26	−6.32	−767.68
8	p-Coumaric Acid	−6.31	−37.59	−6.31	−767.32
9	p-Coumaric Acid	−6.45	−34.56	−6.45	−767.30
10	p-Coumaric Acid	−6.24	−37.08	−6.24	−767.24
11	Ciprofloxacin	−4.31	−59.19	−4.36	−760.89
12	Ciprofloxacin	−3.25	−42.74	−3.30	−759.56

**Table 2:** ADME properties predicted for the phenolic compounds and drug standard.

Properties	Sinapic Acid	p-Coumaric Acid	Ciprofloxacin
Molecular formula	C_11_H_12_O_5_	C_9_H_8_O_3_	C_17_H_18_FN_3_O_3_
Molecular weight (g/mol)	224.21	164.16	331.34
Bioavailability score	0.56	0.85	0.55
Water solubility	Soluble	Soluble	Moderate
Lipophilicity (ilogP)	1.63	0.95	2.24
GIT absorption	High	High	High
Hydrogen bond acceptors	5	3	5
Hydrogen bond donors	2	2	2
Lipinski’s rule	Yes	Yes	Yes
CYP1A2	No	No	No
CYP2C19			
CYP2C9	No	No	No
CYP2D6	No	No	No
CYP3A4	No	No	No

**Table 3:** Predicted toxicity results of the lead compounds.

Compounds	LD_50_ (mg/Kg)	Hepatotoxicity	Carcinogenicity	Immunotoxicity	Mutagenicity	Cytotoxicity
Sinapic acid	1190 (class 4)	Active	Inactive	Active	Inactive	Inactive
p-coumaric acid	1190 (class 4)	Active	Inactive	Active	Inactive	Inactive
Ciprofloxacin	2000 (class 4)	Inactive	Inactive	Inactive	Inactive	inactive

**Table 4:** Minimum inhibitory concentration (MIC) and minimum bactericidal concentration (MBC) of the tested compounds against bacterial cultures.

	Compounds					
Bacterial Strains	Sinapic Acid		p-Coumaric Acid		Ciprofloxacin	
	MIC (μg/mL)	MBC (μg/mL)	MIC (μg/mL)	MBC (μg/mL)	MIC (μg/mL)	MBC (μg/mL)
*Staphylococcus aureus* (Gram-positive)	31.25	62.50	31.25	62.50	7.81	15.63
*Escherichia coli* (Gram-negative)	125.00	250.00	62.50	125.00	15.63	31.25

**Table 5:** The minimum inhibitory concentration and fractional inhibitory concentration index (FICI) of the phenolic acid/antibiotic combination against the bacterial strains.

	Bacterial Strains	MIC (μg/mL)		FICI
Treatments		Sinapic Acid	Ciprofloxacin	
Sinapic acid + ciprofloxacin	*S. aureus*	1.9	0.4	0.3
	*E. coli*	2.6	7.8	0.6
p-coumaric acid + ciprofloxacin	*S. aureus*	7.8	1.9	0.5
	*E. coli*	3.9	0.9	0.3

## Data Availability

Data is contained within the article or supplementary material.

## Supplementary Materials

The following supporting information can be downloaded at: https://www.mdpi.com/article/10.3390/molecules27082601/s1, Figure S1: Timeline plot of all the contacts (top panel) and specific contacts Sinapic acid makes on amino acids of NorA (bottom panel). Figure S2: Timeline plot of all the contacts (top panel) and specific contacts p-coumaric acid makes on amino acids of NorA (bottom panel). Figure S3. Timeline plot of all the contacts (top panel) and specific contacts Ciprofloxacin makes on amino acids of NorA (bottom panel). Table S1: The binding scores of shikimate pathway-derived phenolic acids and NorA efflux pump.
