# Supplementary material for: Computational Insights and In Vitro Validation of Antibacterial Potential of Shikimate Pathway-Derived Phenolic Acids as NorA Efflux Pump Inhibitors
Source: Molecules. 2022 Apr 18;27(8):2601. doi: 10.3390/molecules27082601 (PMC9031328; doi:10.3390/molecules27082601)
Supplement: Supplementary file 1 [file molecules-27-02601-s001.zip › molecules-1580421-supplementary .pdf]

Supplementary materials

Table S1: The binding scores of shikimate pathway-derived phenolic acids and NorA efflux pump.

Protein	Ligands/phenolic acids/standard	Docking score kcal/mol
NorA (PDB ID: 1PW4)	Sinapic acid	−9.04
	p-coumaric acid	−6.91
	Salicylic acid	−5.81
	Benzoic acid	−5.33
	Gallic acid	−5.17
	Syringic acid	−4.98
	Vanillic acid	−4.74
	Caffeic acid	−4.42
	Ferulic acid	−4.25
	p-hydroxy acid	−4.21
	Veratric acid	−4.13
	Isoferulic acid	−5.76
	Protocatechuic acid	−5.07
	Gentisic acid	−4.98
	Umbellic acid	−5.32
	m-coumaric acid	−4.70
	0-coumaric acid	−4.91
	Ellagic acid	−5.23
	Olivetolic acid	−5.84
	B-resorcinilic acid	−5.16
	Orsenillic acid	−4.88
	Hypogallic acid	−5.02
	Ciprofloxacin (standard)	−4.31

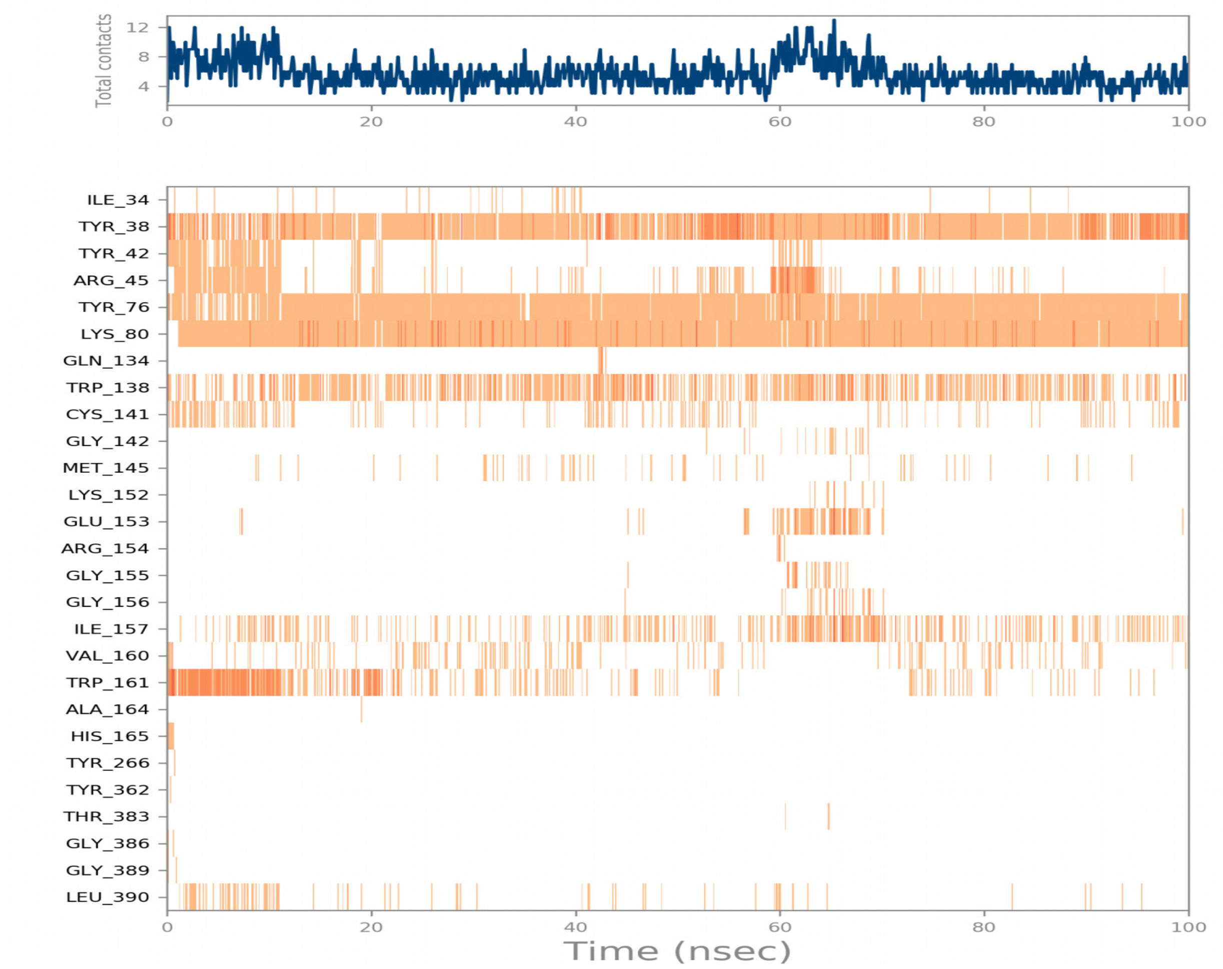

Figure S1. Timeline plot of all the contacts (top panel) and specific contacts Sinapic acid makes on amino acids of NorA (bottom panel).

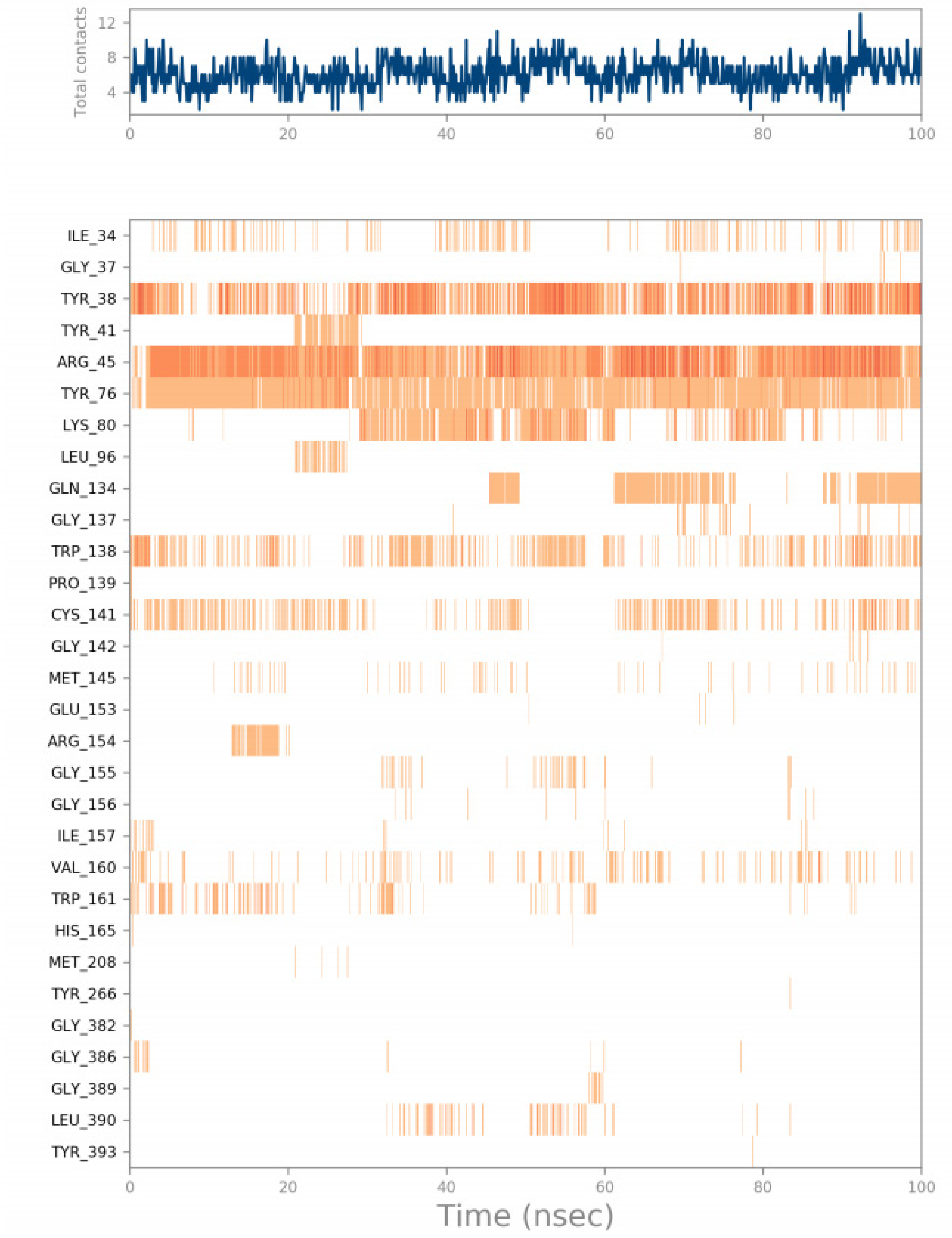

Figure S2. Timeline plot of all the contacts (top panel) and specific contacts p-coumaric acid makes on amino acids of NorA (bottom panel).
